# The Policy of Compulsory Large-Scale Food Fortification in Sub-Saharan Africa

**DOI:** 10.3390/foods13152438

**Published:** 2024-08-01

**Authors:** Victoria Bell, Ana Rita Rodrigues, Jorge Ferrão, Theodoros Varzakas, Tito H. Fernandes

**Affiliations:** 1 Faculty of Pharmacy, University of Coimbra, Polo das Ciências da Saúde, Azinhaga de Stª Comba, 3000-548 Coimbra, Portugal; victoriabell@ff.uc.pt (V.B.);; 2 REQUIMTE/LAQV, Group of Pharmaceutical Technology, University of Coimbra, Polo das Ciências da Saúde, Azinhaga de Stª Comba, 3000-548 Coimbra, Portugal; 3 Vice-Chancellor Office, Universidade Pedagógica de Maputo, Rua João Carlos Raposo Beirão 135, Maputo 1000-001, Mozambique; ljferrao@icloud.com; 4 Food Science and Technology, University of the Peloponnese, GR-22100 Kalamata, Greece; 5 CIISA—Centre for Interdisciplinary Research in Animal Health, Faculty of Veterinary Medicine, University of Lisbon, 1300-477 Lisbon, Portugal

**Keywords:** poverty, food supplements, undernourishment, childcare, premix

## Abstract

Food fortification with micronutrients was initially justified in developed countries by a lack of availability of micronutrients in staple crops, mainly due to soil exhaustion. However, in Sub-Saharan arable lands, soil fatigue is not predominant, and communities consume mostly home-grown, organic, non-processed crops. Sub-Saharan food systems are nevertheless deeply entwined with food insecurity, driver of illnesses. Family production can promote subsistence, food stability, and self-sufficiency, the main SSA setback being the vicious cycle of poverty and the lack of dietary variety, contributing to malnutrition. Poverty reduction and women’s education are significant strategies for reducing child and adolescent undernourishment. Fortification of foods consumed daily by individuals makes sense and can minimize, if not entirely, eliminate deficiencies. Compulsory mass fortification of foods in Sub-Saharan Africa (SSA) with single micronutrients is, however, controversial since they work in synergy among each other and with the food matrix, for optimal absorption and metabolism. Since the causes of malnutrition are many, caused by diverse, unequal, and unjust food distribution, interrelated with political, social, cultural, or economic factors, education status of the population, season and climatic changes, and effectiveness of nutrition programs, just food fortification cannot solve the composite of all these elements. Further, compulsory fortification is excessive, unproductive, and likely harmful to human health, while many challenges remain in assessing the quality of available premixes. Furthermore, aiming at dietary diversification is the best approach of increasing trace element intake from commonly accessible and easily available food sources.

## 1. Introduction

Food production has changed over the decades and is still under permanent improvement, namely in productivity rates of most food crop and cash crop value chains. The current structure of the Sub-Saharan food systems lies at the center of a network of global problems, stretching from poverty to environmental degradation. 

The necessary increase in food production and productivity required to meet future demands cannot be attained by continuing with the existing directions of growth and intensification, since it will undermine the particular support around which the food systems revolve [1]. There is, meanwhile, no possible sustainable population growth or development without social justice and the end of undernutrition, famine, and poverty [2]. 

Over the last half century, in general, the agricultural intensification of staple crops—mainly maize and wheat—has escalated, required to feed the growing and increasingly demanding human population, taking place in different forms, principles and concepts, and norms and values to be effective forms of sustainable and ecological intensification [3]. The most salient feature of sustainable intensified agrifood systems is the greater yield per unit land and time, but this may originate hidden environmental, health, and social overheads and benefits only the large food production chains, not the smallfarmer [4].

Food, not nutrients, is the unit in nutrition, and food diversity and food synergies are determinants for overall health and wellness [5]. Moreover, over 75% of the world’s food production depends on family farmers, representing the vast majority of farming worldwide, both in developed and developing countries. In low income SSA countries, people indeed consume monotonous diets, have a less resilient food system, and are prone to nutrient deficiencies [6]. 

In SSA, food systems have not been suitably accompanied by adequate interventions on access to water, sanitation, and hygiene. Globally, some 2.5 billion people (32%) do not have access to clean and safe drinking water, and approximately 3.6 billion people (46% of the world’s population) lack adequate sanitation services [7]. 

Food and drinkable water affect almost every SSA citizen. Consequently, food and water access, and their quality, are key issues in African human development. A society cannot be considered as developed if these issues are not primarily resolved, since they affect public health, the welfare of people, and human capital, thereby affecting people’s capacity for development [8].

Indeed, the triangle food–water–hygiene insecurities are complex, multi-dimensional phenomena that entail more than availability and access [9], and the present disruptions of supply chains, food, and energy markets have impacted the long-term effects such that the future remains uncertain [10]. Indeed, within the context of the current and consecutive world crisis, sharing common economic, social, climatic, and poly crisis, it is indispensable to consider and react in relation to global food systems, namely cereals. 

Globally, no country can produce all the foods that it needs. Food production has been enough for humankind, but distribution has been inadequate as a food system that should feed every person, every day and everywhere, does not exist. At least 90% of the rural population in SSA, where food insecurity has steadily risen, depends on agriculture as its primary source of food, income, and survival [11]. 

The World Bank, International Monetary Fund (IMF), OPEC Fund for International Development (OFID), and United Nations (UN) have established global targets as part of the Sustainable Development Goals to reduce poverty and “end hunger by 2030”—“the not-so-impossible dream”. While globally there has been some progress in past decades, this target is far from being reached, mainly in SSA, where standards of living are still very low, and chronic poverty is widespread with negative impacts on family dynamics [12]. 

Food insecurity in SSA must be tackled from a multidimensional perspective, beginning with governmental spending on education and health, presently growing very slowly in most SSA countries [13]. Another core problem in SSA has been corruption, abuse of power, and inefficiency of competent authorities, leading to ineffective resource management. Most of the revenues from the exploitation of natural resources (e.g., oil and gas, coal, gold, precious stones) and agricultural staples (e.g., sugar, coffee, cocoa, vegetable oil) fall into political party elites, and national income is not properly distributed to the lower classes, raising the paradox of “poverty in the midst of plenty”. 

The global assessment of the state of food security and nutrition presently reveals that over 900 million people of the 8 billion people in the world, almost one in eight, were suffering from hunger or, more formally, chronic undernourishment, with the highest rates in SSA [14]. 

The population needs access to quality and safe nutritious food ingredients, along with access to health services and a safe environment [15]. 

### 1.1. Some Challenges in Sub-Saharan Africa

The SSA food systems supply most of the food for its growing population (1.2 billion) and have revealed some regions with high levels of resilience and others with significant vulnerabilities to specific shocks, depending on the global markets and export-oriented cash crops (e.g., cotton, cacao, tea, and palm oil) [16]. 

Bringing back neglected crops (e.g., sorghum and millet; kale, eggplant; algae), displaced by crops better suited to commercial farming, is important to increase the number of food options and diversifying markets, while building resilience to climate changes. For example, finger millet (*Eleusine coracana*), fonio (*Digitaria exilis*), and teff (*Eragrostis teff*), have higher iron contents than maize, rice, and wheat [17]. 

Despite many attempts by multiple NGOs striving to sustain positive outcomes, by building local resources and capacity, assisting attitude changes, and strengthening the environment to save lives, improve health, build resilience, increase economic productivity, and advance development [18], the fact is that SSA’s family-based agriculture small-scale food chains’ actors and small millers do not have the regular access to funding and production inputs to attend to the present mandatory legislation and prohibitive costs of premixes, technological demands, and staff training [19]. 

The Gates Foundation has been supporting large-scale food and certain vehicles of fortification programs in some 29 countries in SSA since 2008, through grants to GAIN (Global Alliance for Improved Nutrition) and UNICEF [20]. Although the African Union had declared 2022 as its “Year of Nutrition”, many questions remain as to whether the national statistics are reliable enough to enable proper decision-making on food and beverage fortification, and how to implement the compliance to standards among the millers and suppliers of premixes in SSA [20]. 

Most SSA countries, unseen in developed countries, have produced harsh legislation for food processors and millers on the compulsory fortification of maize, wheat, cooking oil, sugar, and salt, initiatives propelled by foreign pressures and national lawyers’ offices for business purposes but with no scientific support on assessments of African foods [21]. 

Another significant and ignored question is the potential socioeconomic effects concerning the risk of competition between large-scale and small-scale processors, and the possible adverse effects on the sustainability of poor smallholder family farmers and communities [22]. 

There is still the question of whether and how the mechanisms of mixing foods and the delivery of premix supplementation and vehicles can benefit/jeopardize domestic food chains family producers and generate overall socioeconomic development locally [23]. 

Food-based strategies to combat micronutrient deficiencies have been absent, which does not sustainably solve the micronutrient deficiencies but enriches a few actors. Enforcement for fortification should focus on the origin of bulk items by large-scale producers and never the small miller [24]. Present foreign aid and other support does not directly reach the small producer and is used to cover the premix costs and the unnecessary national committees coordinating and monitoring the fortification activity, under high fines by national authorities to the non-compliant. 

Fortification could be an effective medium-term strategy to tackle nutritional risk in vulnerable populations in some contexts but, after more several decades experience, it can be seen to have carried negative environmental, economic, and social impacts [25]. Dietary diversification, on the contrary, is known to be a sustainable way to overcome micronutrient deficiencies, bringing long-term benefits, including nutritional ones, and the provision of ecosystem services [26]. 

Nutrition performance needs to be linked with sustainable agricultural systems thus strengthening the agro-biodiversity of resilient cropping systems. This would enable the strengthening of the link between agro-biodiversity, aquaculture systems, and food diversity [27].

In the framework of the SDGs, the EU–SSARI Strategic Partnership on FNSSA proposals describe how projects can contribute to sustainable, healthy African diets. The research, innovation, and action of the EU project, InnoFoodSSA (2023), focuses on nutrition, with the aim to improve the nutrition and well-being of African people via the analysis of diets. A survey was conducted on Sub-Saharan diets with the example of aquaculture and fishery industries managed in SSA guaranteeing food security and economic growth [28,29,30,31].

### 1.2. The Global Supply and Use of Premix

The social injustice behind our food systems, from growing and harvesting to procurement, is serious and lasting. Basically, the United Nations FAO, the hundreds of nutritional aid/assistance organizations, and even SSA governments, have been the subject of a “corporate capture” of the global and national food and nutrition policy spaces, where undernutrition has been separated from its social, public health, economic, political, and cultural causes, not addressing the basic questions [32]. 

This may be exemplified by the failure of the “Green Revolution”, which sought to halve hunger in 20 African nations and double the income and yields of over 30 million small-scale farmers by 2020. However, instead of delivering on its promises, it appears that hunger has actually escalated by 30% in these countries [33].

The pharmaceutical industry, because of possible hazards to a patient’s life, is the industry more highly regulated in the world; however, vitamins and trace element premixes to be included in diets are not included in this scrutiny [34]. The role of a premix producer may be simple mixing, pelleting, microencapsulation, delivery, or some other form of activity before delivering the final form of the product to the manufacturer or brand owner.

The example of wheat flour fortified with folic acid shows the interpretation of the “minimum” content as the average addition by the industry. The problem arose from the premix formulation because the instructions for the addition were being followed by the millers based on what they had received from the manufacturers. Hence, misinterpreting the minimum content as the average content, the program delivered half the amount of folic acid that had been planned to be provided [35]. 

In Chile, there have been variations in the fortification and more than 50% of samples were either below or above the regulatory range. In the U.S., an average of 100 μg/day of folic acid was provided with a minimum content of 1.4 mg/kg for cereal flours [35]. Hence, this content was selected as a basis for attaining benefits for those in need without potentially risking the excess intakes that may go beyond the tolerable upper intake level (UL) [36]. 

Most SSA governments have produced food fortification mandatory laws, without pilot studies, in partnership with external “non-profit” groups clearly associated with the premix industry, and with no precautionary principles such as caution against consuming iron-fortified food by patients with thalassemia and sickle cell anemia [37]. 

There is a clear global need for a new intervention framework, which is very difficult to achieve due to market cartels, presently governed by huge corporations, some even in the past guilty of participating in a worldwide conspiracy to raise and fix prices for vitamins [38]. 

The Dutch DSM Nutritional Products, the world’s largest supplier of vitamins, carotenoids, and other biochemical products, having acquired Swiss Roche Vitamins, is the main world manufacturer of nutritional and pharma ingredients. For enzymes and trace minerals, Cargill Incorporated (Minneapolis, MN, USA), ADM (Decatur, IL, USA), BASF SE (Ludwigshafen, Germany), Bluestar Adisseo Co., Ltd. (Shanghai, China), Unilever (Rotterdam, The Netherlands), and many others, are in control of the markets. Several “non-profit” organizations in SSA have present or past commercial links with some of these [39,40]. 

Umbrella groups or coalition of the NGOs, significant international consortium partners since 2002, GAIN (Global Alliance for Improved Nutrition) and FFI (Food Fortification Initiative), public, private, and civic sector partnerships, have been referred to as lobby groups for vitamins, minerals, and nutraceuticals, operating their own premix facilities to produce and supply fortified foods to the SSA markets [41]. 

It must be noted that the WHO (World Health Organization)’s standing committee on non-governmental organizations (NGOs) deferred the accreditation of Swiss-based GAIN as an NGO, raising the issue of conflicts of interest in the NGOs’ ties with businesses. GAIN’s vision is tied to fortifying food with “missing nutrients” with the help of global food companies and selling it to people “at low cost”.

Based on publicly available information, national regulatory bodies include private advantages from corporate actors, many domestic and international NGOs functioning as pressure groups, some with predatory tactics to advocate for fortification-friendly policies in SSA and other parts of the world, with national authorities not preventing this conflict of interest [42]. 

Although there is no recommended daily intake figure, FDA and EFSA suggest guidelines of tolerable upper intake levels for vitamins and minerals considered to be both safe and sufficient [43]. 

The global food premix market is a massive business valued at USD 6.31 billion in 2023 and estimated at USD 10.7 billion by 2029, and this is projected to consistently increase. The global animal feed premix market, segmented into amino acids, vitamins, minerals, antibiotics, antioxidants, blends, and others, is even higher and expected to reach USD 33 billion in 2025 to maintain the global sustainability of livestock, poultry, and fish production [44]. 

While in intensive animal production systems, the need for premixes is easier to calculate through feed conversion efficiency data; in humans, there is no single best test to evaluate the nutritional status, and the nutritional requirements of healthy individuals depend on various factors, such as type of diets, age, sex, and activity [45]. Furthermore, to date, there are no precise data on the requirements of most micronutrients for individuals or communities, and the recommended values of dietary intakes strongly vary for each group of individuals [46]. 

Large-scale food fortification programs face one of the most significant recurring input costs, and that is vitamin and mineral premixes. Despite some noteworthy innovative approaches for improving the access to quality vitamin and mineral premix in fortification initiatives [47], a number of barriers persist in SSA in the absence of national quality assurance, monitoring of delivered products, and the absence of funds to purchase them.

Instead of pushing mandatory food fortification of doubtful benefit and potential harm, and insisting on unsustainable, uncertain, and expensive procedures of large-scale mass food fortification, there is the need to launch a major awareness campaign to improve SSA’s food and nutrition security, by the replacement of refined, mostly imported wheat for pleasant bread products, shifting to more realistic, nutritionally balanced diets, increasing economic revenues and equitable benefits, and improving the sustainability and resilience of the SSA food system [48]. 

The difference in food preferences and their content in micronutrients are often not taken into account to measure the undernourishment of SSA populations. Presently, only limited food availability data (estimates made only for few commodities) exist, from production and trade, and the use of consumption levels across the populations. This is the basis for an unjust estimation of the proportion of the SSA communities that is unable to meet their daily nutrient requirements [49].

Although these assessments are, in essence, inaccurate and vague, with many valid criticisms against them, in the absence of better indicators, they are used extensively to monitor food security status [50]. Only local studies based on either real food analyses or traditional behavior may provide genuine data. Lack of knowledge of foreign experts on the use of traditional foods, herbs, condiments, and medicinal plants, and estimation methods leads to the overestimation of malnutrition percentage levels in SSA [51]. For instance, estimates of the undernutrition prevalence, namely in pregnant women, could vary a lot and range between 5 and 18% and 14 and 48% for the same populations, as reported in different studies [52].

These developments have helped the further ‘medicalization’ of nutrition, which has presented donor agencies with simplistic, ‘magic bullet’ product-based solutions, such as food fortification, to malnutrition [53]. 

Another claim is that the growth impairment experienced in the first year of life is very difficult to overcome in the later years of childhood. The fact that an individual child has a short stature and falls below the 5th percentile on a growth reference curve may reflect a normal variation in growth within a population. An individual child may be short simply because both parents carried genes for tininess and not by virtue of deficient nutrition [54,55].

The SSA countries with the largest frequency of impaired growth and development are found in the southeastern regions, reflecting a complex set of challenges that include civil conflict, economic downturns, commodity price shocks, and droughts and floods, or the legacies of such events [49].

It is important to observe that food production is not the limiting element in many of these hunger hotspots of malnutrition, and a multiplicity of factors including socio-cultural perspectives are deeply involved, along with an unfair access to food [56].

Unsuitably, SSA governments are often presented by the United Nations and premix manufacturers with an imaginary consensus on the way to solving malnutrition that emphasizes the role of the private sector and the need to include fortification of food in policy formulation.

The aim of this paper is to discuss fortification in SSA and the causes of malnutrition which are mainly due to diverse, unequal, and unjust food distribution, interrelated with political, social, cultural or economic factors, education status of the population, season and climatic changes, and the effectiveness of nutrition programs, highlighting the innovation of this work.

## 2. A Brief History of Food Fortification

The industrial fortification of widely consumed foods was developed as a strategy to prevent and reduce the prevalence of specific nutritional deficiencies as a consequence of the limited availability and affordability of an adequate diversified diet, that includes plant- and animal-source foodstuffs [57].

The WHO defines food fortification as the practice of a deliberate increase in the content of one or more micronutrients (i.e., vitamins and minerals) in a food or condiment to improve their nutritional quality, thereby providing a public health benefit with minimal risks [58].

The earliest food fortification was suggested by a French chemist in 1820, with a proposal of fortifying salt with iodine, and in Switzerland in 1923, and the United States in 1924, when iodine was primarily combined to table salt on an optional basis, in an effort to manage the widespread health problem of goitre [59].

The sourcing of foods from many diverse locations has essentially eliminated the problems of goitre caused by an iodine deficiency in foods from certain geographical areas, and attention is presently given to pregnant women and women of childbearing age, who may be at risk for mild to moderate iodine deficiency [60].

Fortification is usually carried out by public or private stakeholders, aiming to provide public health protection from deficiencies with micronutrient premixes supplied by several mega-corporations, which are added mainly to flour in the industrial milling process. They should not affect the taste, smell, texture, or baking qualities of the end product.

**Figure 1:**
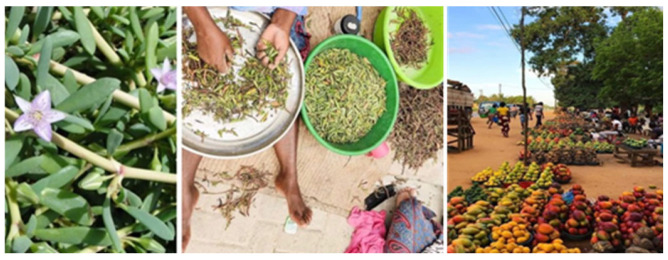
A sprawling perennial herb (*Sesuvium portulacastrum*), from the family *Aizoaceae*, grows in coastal and mangrove areas throughout the world, is a staple food and typical dish (“*sirisiri matapa*”) in the north of Mozambique. Tropical fruits are abundant.

In the SSA communities, people eat considerable amounts of leafy greens, manioc tubers and sweet potatoes, local beans of all kinds, nuts (e.g., cashew, coconut), seeds (e.g., sesame), and peanuts, fish and seafood, herbs and spices (Figure 1) [61,62]. Only on special occasions would they then eat meat and sweets. This scenario is probably superior when compared to developed countries where ultra-processed fast foods and fizzy drinks prevail and overweight/obesity is very high [63,64]. African native diets hold potential nutrients and foods that may be beneficial in protecting them from having chronic diseases including cancer, as opposed to the ‘Western diet’ that has increased the risk for multiple types of cancers. There is, however, a need for large cohorts studies with comprehensive datasets for effective research [65].

In the poor rural regions of SSA, despite the minor levels of ultra-processed foods, micronutrient malnutrition (or hidden hunger) exists namely when there is undernutrition caused by crop shortages. This is becoming common in cases of a lack of diet diversification [14].

While SSA governments have taken several steps to address these issues, food fortification has emerged as a widely discussed solution to tackle malnutrition. However, it is not only malnutrition but also non-communicable diseases (NCD) being an equivalent burden [66]. 

Food enrichment is the practice of adding micronutrients that might have been lost during picking or processing back into a foodstuff, while fortification adds additional micronutrients absent (or present in minor amounts) prior to processing [67]. Increasing the levels of essential vitamins and minerals in growing staple crops that are widely consumed by people in affected communities such as in SSA is another sustainable strategy named biofortification, which addresses micronutrient deficiencies [68,69].

Large-scale public health programs with single nutrients were perceived to have a poor impact on the target health desired outcomes, both in Asia and in Africa, as seen on the dormant reaction of the frequency of anemia to ongoing iron supplementation programs, for example [70].

Indeed, to be really successful, local settings, sociocultural influences, and environmental conditions should be considered as key determinants for enhancement of the micronutrient status of populations, all of these factors presently absent in SSA [71]. Moreover, the dietary characteristics of the population should be taken into account in order to select the adequate fortification condition with the highest effectiveness potential [72].

This is not performed or managed adequately and routinely and, recurrently, it is considered as a freestanding diagnosis. Furthermore, deficiencies in blood are, in fact, clinical signs which show an underlying etiology. However, elucidation of its cause is required. Clinical discordance in both the formal definition of deficiencies and in screening protocols is clearly shown [73].

Fortification with micronutrients is generally considered valuable in the appropriate dose, but conceivably detrimental when ingested in excess [70,71,74].

## 3. Global Large-Scale Food Fortification

Despite being mandatory in most SSA countries, food fortification in the United States of America has evolved tremendously over the years and is not generally compulsory; the FDA made a decision in the 1940s that it would not require mandatory fortification for any food product. This policy is still in place and, with exception to wheat flour, enrichment is not a requirement generally speaking, but if enrichment/fortification is claimed then there are standards that must be met [75]. 

In Canada, some sectors of the food industry and consumers see the current regulatory controls on the addition of vitamins and minerals to foods as overly restrictive [76,77]. 

Food consumption varies widely among countries and is influenced by many factors, including social, economic, cultural, environmental, educational, health-related, and physiological elements [78]. The European Union has published, by commissioning many interested parties, connected with cereal milling and micronutrient suppliers, legislation on fortification of foods and condiments, without mentioning how to measure the outcomes and evaluate its contribution to improving public health objectives [79]. 

Food fortification is not prohibited by the laws or regulations of the European Union, as long as certain requirements are fulfilled, such as the minimum and maximum amounts of the nutrients added. Fortification is not allowed for alcoholic beverages and unprocessed foods, and adding vitamins and minerals to food is permitted but not compulsory in the European Union through regulation 1925/2006. Many EU member states have mandatory fortification legislation, but it is possible that the extent of food fortification has changed over time, also in the absence of clear fortification policies, which makes up-to-date summaries difficult. 

Foods intended for specialized nutrition, namely foods for infants and young children, foods for calorie-restricted diets, and dietary foods, are not considered voluntarily fortified since they are covered by specific legislation [80].

The United Kingdom has a distinct large-scale mandatory program for the fortification of wheat flour with iron and other key nutrients since the 1940s. In the UK, the Bread and Flour Regulations (1998) have laid down labeling and compositional standards for bread and flour; they have also specified that calcium, iron, thiamine (Vitamin B1) and niacin (Vitamin B3) must be added to all white and brown flour. Large-scale mandatory fortification of wheat flour and vegetable oil exists in Burkina Faso, Cameroon, Côte d’Ivoire, Mali, Nigeria, and Senegal [81].

In India, the fortification of foods is not compulsory, except for iodized salt, with plans to make fortification mandatory for packaged edible oil and milk, pushed by corporate classes which stand to benefit economically from fortification in India and after alleged flagrant conflicts of interest in official regulation bodies. However, with many criticisms against mandatory food fortification, with the decision-making body remaining opaque, pushes for iron fortification of rice is still a topic of debate [32].

In China and Mongolia, where the prevalence of multiple micronutrient deficiencies has decreased substantially in recent decades, despite pressure from the global micronutrient industries, food and beverage fortification has been mandatory only for iodized salt and, on a voluntary basis, for some 50 types of products [82]. A policy of large-scale industrial fortification of wheat flour and wheat flour products, edible oil, and milk has been in place in Mongolia since 2018 [83].

The picture is somewhat different in the SSA region, where the livelihoods of small-scale producers and processors have been swiftly threatened by stringent mandatory regulations, varying with the food vehicle, and difficult to implement even by local regulatory authorities. 

A sharp fortification program can only be responsible, effective, and sustainable if there is an adequate knowledge of the target foodstuffs and diet composition, and an accurate control of micronutrient deficiencies, both yet impractical in most SSA countries.

In developed countries where food supplements are regulated as foods, directives exist for the marketing and labelling of both fortified foods and supplements, namely for individual use by athletes [84]. In SSA, a totally different objective of large-scale food fortification is observed, where legislation on compulsory fortification has been unaccountably established. 

In SSA, many hundreds of national, regional, and global organizations with assorted designations and agendas are well established, created in support of private or public stakeholders to improve the supply of missing nutrients (e.g., vitamin A, thiamine, riboflavin, niacin, pyridoxine, folic acid, iodine, iron and zinc) in developing countries [85]. 

However, despite the implementation of national regulations to fortification of foods in some 29 African countries and in other continents, foisted by corporate-led premix suppliers and not backed by adequate dietary indigenous studies, the procedure has been conducted without the necessary pre-evaluation on the composition of local native foodstuffs, plants, and type of diets, gut microbiota profile, specific for each regional location. 

This development aid/assistance obviously covers the economic interests of these “non-profit” organizations, which reap direct or indirectly colossal benefits, with no follow-ups on the outcomes of such programs or the design of guidelines for effective intervention public health strategies [86].

Nevertheless, despite the lack of adequate epidemiological studies for each region or population, it is commonly generalized that there are deficiencies in micronutrients such as iodine, iron, zinc, and vitamin A. These are usually diagnosed by specific symptoms in an ambulatory clinic and are common in developing countries, compromising the physical health and cognitive abilities of millions of people [87]. 

However, in SSA countries, the dietary assessments necessary to ensure adequate complementary nutrition and hydration intake has been absent, therefore food fortification has been conducted in a bemused manner but with good intention. After more than three decades of implementation, the science underpinning the policy for micronutrient addition to foods continues to evolve, but in SSA, in our view, there is the need for a more flexible framework.

We have surmised that nutrient requirement values are just guidelines and not fixed figures [46], and it must be noted that the micronutrient balance is determined by complex mechanisms, including those not taken into account in the past, such as gut microbiota modulation specific for different populations [88]. Moreover, micronutrient deficiency measured only by serum/plasma levels is not adequate when utterly associated with dietary inadequacy, varying according to individual and community characteristics [89,90]. 

In Africa, the justification of soil overuse for fortifying staple foods is neither acceptable nor evident, and degradation and restoration are not the main causes, although deforestation may be a cause on some occasions. Forests, or better, large trees, are continuously destroyed in the search for energy in the form of firewood, and for timber sales to developed countries, causing increased environmental damage and soil erosion [91].

Despite the poor definition of agricultural sustainable intensification [92], in all situations, novel approaches and practices to sustainable agriculture in different regional models are required in order to apply innovative technologies and principles, namely in heterogeneous and marginal lands [93]. 

Sustainable food systems for food security in SSA needs a combination of local and global approaches, considering the links between food security, access to land and natural resources at the local level, handling of losses and wastes, and flexible food value chains, while observing the unpredictability and risk in the markets and the supply chain [94].

## 4. Micronutrient Deficiencies

The reality is that most African countries lack adequate evidence of the populations’ nutritional status, knowledge of the population consumption patterns, adequate laboratory facilities and trained staff for micronutrient evaluation, permanently facing the challenge of determination of the levels of nutrient deficiency and the need for supplementation. 

Multiple vitamin and mineral statuses are rarely measured in rural and semi-urban areas of SSA, even in group risks such as pregnant women. In a semi-urban area of Ghana, the relatively low prevalence of individual micronutrient deficiencies and the micronutrient status were not related to common blood-based health biomarkers, leading to co-occurring and overlapping of multiple deficiencies [95]. 

Since diet-related micronutrient deficiencies are concurrent with multiple micronutrients, food and beverages fortified with single micronutrients have been considered fragile as only coordinated multi-micronutrient programs may combat the co-existing deficiencies [96]. However, since the statuses of the general and high-risk populations are not monitored on a regular basis, eventual deficiency diagnosis is not normally diagnosed in the SSA communities, but it is instead compelled by the hundreds of organizations supplying nutritional indications or means of fortification, with the most prominent “non-profit” having strong links to premix megacorporation producers [97]. 

Fortification of staple foods such as rice, maize, and wheat flour in SSA has been reported as a strategy for combating micronutrient malnutrition [98,99]. Moreover, commercial compulsory food fortification has been small-scale in SSA and other developing regions [100,101] due to socioeconomic factors hindering the practice and the cost of commercial fortified food products and official control of the program. In addition, the choice of delivery vehicle may affect the bioavailability of the micronutrients in fortified foods [102].

Since the causes of malnutrition are many, the silent hunger of micronutrient deficiencies continues, based on diverse and interrelated political, social, cultural or economic factors, education status of the population, season and climatic changes, urbanization, family disunity, alcohol intake, prevalence of infectious diseases and the effectiveness of nutrition programs [103], with the evident question being how a food fortification system may resolve these problems [104]. 

Obese individuals show a higher prevalence of micronutrient deficiencies compared to normal-weight people [105], despite them eating more to satisfy their nutritional requirements. In countries where overweight and obesity prevail as a malnutrition manifestation, there is no enforcement of food fortification but this is globally an ongoing debate [106]. 

In most of SSA, the age of the child strongly correlates with increased levels of malnutrition. Newborns in many African households live in an unhealthy environment and, in many cases, receive suboptimal feeding, that is, a lack of exclusive breastfeeding and inappropriate or untimely complementary feeding thus leading to the decline in nutritional status from birth [107]. The first year of life shows a growth impairment and is considered very difficult to overcome in the later years of childhood. But there is no substantial long-term evidence if this is true [108]. 

Fortification is considered one of the most cost-effective interventions that subsist to address micronutrient malnutrition (Table 1). While the South Africa’s food and beverage vitamin-fortified and mineral-enriched market is dynamic and fierce in nature, having a large number of national and megacorporation stakeholders, striving to capture a significant share and competing for market quota, however, this outlook may not be replicated in most other SSA countries in the same manner [109].

African communities consume diets with low content of animal products and a high consumption of vegetables and cereal crops rich in phytates, which may induce zinc deficiency [111]. 

Therefore, compulsory food fortification is debatable and should not be uniform in SSA, where indigenous food composition and gut microbiota studies were never or rarely conducted, which would be necessary for an adequate assessment of micronutrient requirements. This has raised the question of if food and beverage fortification in SSA is based on science or business or both [112]. 

Available data concerning malnutrition in SSA derive mainly from international organizations that calculate data, rather than being determined by national observations or statistics. Locally conducted nutritional trials are practically nonexistent, so malnutrition levels are inferred indirectly from agriculture production, foodstuff imports, and consumption based on population numbers [113]. 

Another “sophisticated” complex assessment includes the computation of customary dietary crude energy intake levels (Kcal/person/day) for the average individual, the probability distribution, and the modeling of a parametric probability. The precision of the estimates is considered generally low due to the probabilistic nature of the inference and the margins of uncertainty associated with the variables in the model [14]. 

Even FAO bases their data by comparing the estimated dietary energy intake with the average energy requirement standards to measure food deprivation and undernutrition. However, this factorial methodology estimates the population means or totals on energy requirements but does not take into account micronutrients and the actual individual requirements at different physiological stages, which are unknown [114].

Furthermore, this may not adequately reflect the African picture, and awareness must be evidence-based and built on data that accurately and completely capture the occurrence, causes, prevention, and treatment of malnutrition in all African sub-populations [115]. These data are critical for researchers, clinicians, non-governmental organizations, ministries of health, and other policymakers to prioritize efforts that address SSA’s malnutrition burden. Furthermore, it is important that all stakeholders are represented in the data collection and application, taking into account the effect of ecological area into the dietary patterns of the African community and the specificities and food habits of each region [116]. 

Sustainability in health can be achieved with the maximization of the connections between ecological systems and food systems, hygiene–sanitation, food security, and safekeeping. None of these is sufficient by itself, and all are necessary for high-quality human development [117]. 

## 5. The “Big Four” Micronutrients

Daily Reference Intakes (DRIs) for most micronutrients (vitamins and trace elements) have been established in the USA by the federal government’s 2020–2025 Dietary Guidelines for Americans [118], but not in SSA, where the WHO guidelines are followed [97,119]. 

It must be emphasized that some of these micronutrients may interact with bioactive food components capable of forming insoluble complexes (e.g., phytates) and medications [120]. 

### 5.1. Iodine

Globally, some 2 billion individuals have insufficient intake of iodine, and around 50% of the adult population in Western developed countries are iodine-deficient [121]. Iodine in the thyroid gland participates in a complex series of reactions to produce thyroid hormones. The average adult only needs some 150–250 μg/day, which can be supplied by less than half of a teaspoon of iodized salt. Nevertheless, about half of the manufacturers of salt no longer add iodine to their product because foods from other regions (e.g., of marine origin) have high natural iodine levels, and sourcing them has essentially eliminated the problem [122]. 

### 5.2. Vitamin A

A review suggested that fortifying staple foods with vitamin A may make little or no difference to the serum retinol levels, a biomarker of vitamin A status [123]. Another case to examine would be a serum/plasma retinol level of 70 μmol/L or less defined as subclinical vitamin A deficiency. No difference would be made there as well. It is not very clear whether a reduction in clinical vitamin A deficiency can be achieved with this fortification [123]. 

Animal livers enclose large amounts of vitamin A, followed by alternative origins such as meats, eggs and some fish [124]. Consumption of 100 g of orange-flesh sweet potatoes would provide 50 µg of Vitamin A [125]. The Recommended Dietary Allowances (RDAs) for Vitamin A have been well established for different physiological stages and ages [126]. It must be noted that a high consumption of vitamin A, leading to hypervitaminosis, has been considered to cause a number of adverse effects and even appears to be a teratogen [127]. 

Orange-flesh sweet potatoes (*Ipomoea batatas*) appear to be a better source of vitamin A, along with β-carotenes, and its use African diets has been labeled as beneficial [128]. Nevertheless, most SSA populations have more access to vitamin A via organ meats that contain a higher percentage of vitamin A than orange-pulp sweet potatoes [129], and rich plant sources (e.g., chili and red peppers, papaya, loquat, mango) of β-cryptoxanthin or its esters [130]. 

Therefore, the poor vitamin A status varies a lot across SSA countries in children aged 2–5 years old, averaging 42%, and supplementation needs to take this into account with the adequate diagnosis, since large doses of vitamin A can cause liver damage [131]. However, addition of vitamin A alone to the staple foods might show little or no effect on vitamin A status or deficiency. Comparison of staple foods fortified with vitamin A versus no intervention has been evident in very few or no studies [123]. 

It must also be noted, before fortification, that low levels of plasma concentration of vitamin A may also be related to onchocerciasis (“river blindness”), a parasitic worm infection transmitted by flies, affecting millions of people in the endemic regions of SSA, and control programs often integrate vitamin A supplementation with ivermectin treatment [132].

Provision of staple foods fortified with vitamin A and other micronutrients does not necessarily increase serum retinol concentration but, apparently, it lowers the risk of subclinical vitamin A deficiency compared to unfortified foods [123].

A comparison of RAE (retinol activity equivalent) among different food sources is shown in Table 2.

### 5.3. Zinc

It is easy to get enough zinc from a healthy diet. Zinc, an important cofactor in the body and naturally present in some foods (e.g., oysters, red meat and poultry, fish, seafood, beans, peanuts, cashew nuts, and whole grains), is involved in cellular metabolism, namely for the catalytic activity of hundreds of enzymes, playing a role in enhancing immune function, protein and DNA synthesis, wound healing, and cell signaling and division [133]. 

Therefore, it is difficult to accurately assess zinc insufficiency or excess [120,134], and successful zinc fortification strategies should consider the impact of zinc bioavailability on the whole diet due to the impact of phytate and protein on zinc absorption. The recommended dietary allowance is 11 mg for men and 8 mg for women. Taking daily high amounts for long periods has been connected with aggressive prostate cancer, warranting precaution of excessive usage of zinc among adult men [135]. 

Zinc can decrease the effectiveness of antibiotics (e.g., ciprofloxacin, tetracycline), antirheumatic drugs (e.g., penicillamine), and blood pressure drugs, but mass fortification usually does not take this into account [136]. Considering the possible toxicity of high dietary intake of zinc, its load and source should be taken into consideration prior to fortification.

### 5.4. Iron

Heme iron is found in meat, poultry, and fish, while non-heme iron is identified in plants, eggs, and nuts. Chronic iron deficiency is the world’s most prevalent mineral deficiency and is the most common cause of anemia among an estimated 2 billion people worldwide [137]. Adults should take no more than 45 mg of iron a day as it can be harmful, since an iron overdose can be toxic thus causing organ damage [138]. The generally recommended dose of ferrous sulfate for children is 3 mg/kg of iron once or twice daily (maximum total daily dose is 150 mg of elemental iron) [139]. 

Biofortification is based on plant breeding to improve the nutritional quality of food by increasing the nutrient content and availability [140]. This approach has proven to have better iron accessibility and bioavailability than fortification, being inexpensive, sustainable and efficient in providing micronutrients for poor populations, thereby complementing conventional interventions [141]. Biofortification also involves the use of biotechnological techniques (e.g., genetic modifications) with transgenic and breeding techniques being employed for the biofortification of cassava, banana, and cauliflower [142,143].

Food-to-food fortification (FtFF) is another strategy involved in the promotion of the bioavailability of essential micronutrients. This is carried out by increasing the content of micronutrients and enhancers of their absorption and decreasing the levels of inhibitors of micronutrient bioavailability [144]. Improvements in iron and zinc bioaccessibility in pearl millet have been carried out with FtFF using moringa leaves and baobab fruit pulp [145], with the same process for maize [146] and similarly for sorghum-based foods where extrusion cooking has helped alleviate iron deficiency by reducing the content of anti-nutrients [147]. Iron levels in African foods are described in Table 3.

## 6. The Link among Poverty, Food Insecurity, and Malnutrition

A reciprocal connection exists between poor nutrition and poverty, creating a vicious cycle with each fueling the other, both serving as the cause and consequence of each other. Undernutrition produces conditions of poverty by reducing the economic potential of the population and, likewise, poverty reinforces malnutrition by increasing the risk of food insecurity [148]. 

Despite the general claims of the United Nations that an increase in extreme rural poverty in developing countries is entirely linked to the global food insecurity crisis [149], in SSA countries, action plans have been approved for family subsistence farming, which have strengthened their resilience and provided effective solutions to deal with the emerging needs of agrifood systems, leveraging local and regional cooperation to accelerate inclusive development and reduce poverty [150]. 

Ending world absolute poverty is an illogical goal, and the chances of ending poverty entirely are zero. But a sustained poverty reduction is a key concern of all those interested in the development of poor countries. Poverty, no matter how it is defined (there is no universal definition), is a relative concept with its multidimensionality thus requiring different policies for poverty reduction, since it is not confined to the material aspects of life, and includes social, cultural, and political aspects as well [151]. 

**Figure 2:**
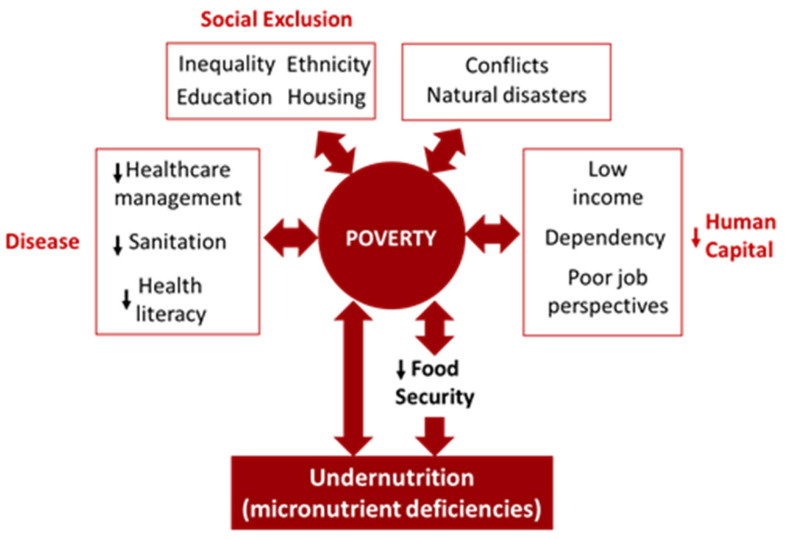
Interdependence among poverty, social exclusion, diseases, human capital, food security, and micronutrient deficiencies.

Solving poverty solely with economic strategies is not realistic, as it involves holistic attention on a set of harmonized initiatives and all-round economic recovery, necessary to increase wages and consumption. Indeed, despite the good economic development in several SSA countries, poverty and hunger continue to persist [152] (Figure 2).

### Mother–Child Malnutrition in SSA

The initiation of breastfeeding within 1 h of birth, and exclusive breastfeeding for the first 6 months, is the best way to give infants the nutrition they need [153], and in order to try and overcome micronutrient deficiencies and coexisting complications in <2 years of age children, food and beverage fortification schemes have been implemented in several SSA countries, together with the continuation of breastfeeding for up to 2 years of age or beyond [154]. 

Fortification feeding practices have revealed a noticeable beneficial effect on the continuous growth of a child within the first 2 years of life; however, there remains no evident direct demonstration of the cause–effect relationship between fortification and growth [155]. 

Child malnutrition is affected by several determinants, such as intrauterine growth restriction, lack of exclusive breastfeeding, inappropriate complementary feeding, and the recurrence of infectious illnesses, food scarcity, and micronutrient deficiencies [156,157].

The most commonly employed indicators of child malnutrition in SSA have been the anthropometric measures of child nutritional status (stunting, underweight, and wasting). However, the most used indicator, the body mass index (BMI), dating back nearly two centuries, is considered an unsound, basic, obsolete, overrated, and inaccurate measure of body fat content and does not take into account muscle mass, bone density, overall body composition, and racial and sex differences, and can result in missed diagnoses [158]. 

Fortification of maize meal and wheat flour involved in bread production is mandatory, while commercial infant products are widely available in several SSA countries [99,159]. This practice of complementary feeding practices of young children has its risks since excessive intakes may remain unacceptable. This child survival strategy for developmental outcomes shows unclear benefits [160,161]. 

The poor health and nutrition of the mother lead to low birth weight, reflecting fetal growth impairment, and serves as an indicator of the risk of infant mortality and future poor health [162]. The burden of undernutrition through the life cycle and across generations, and the persistent transmission of poverty trauma within African communities, remains unacceptably high and is a critical measure [148,163].

## 7. Alternatives to Food Fortification

While fortification is considered a successful short- to medium-term strategy to tackle nutritional risk in endangered communities in some frameworks, dietary diversification, on the other hand, which is rather challenging to implement, is a sharper and more sustainable way to overcome micronutrient deficiencies, with long-term benefits, aside from nutritional ones, with the provision of ecosystem resilience [69].

Originated in the region, indigenous foods are by far superior to introduced exotic foods, and they are also culturally acceptable and adapted to local climatic conditions, having been consumed traditionally for generations [164]. Reintroducing and scaling up neglected crops can be a sustainable food- and climate-smart solution for SSA, by building local and regional markets with public incentives and investments, including in infrastructure development, regulations, and subsidies [165].

As another alternative to individual food fortification, the latest evidence shows that small-quantity (ca. 20 g/sachet) lipid-based nutrient supplements (SQ-LNS) for young children (6–23 months of age) significantly reduce child mortality, stunting, wasting, anemia, and adverse developmental outcomes, and they are considered more cost-effective than other options such as micronutrient supplementation or the provision of complementary food [166,167,168].

SQ-LNSs usually include ω-3 fatty acid-rich vegetable oil (e.g., canola/rapeseed or soybean oil), legumes (e.g., peanut, chickpea, lentil, and/or soy), milk powder, and a small amount of sugar (for palatability). In addition, the formulation is fortified with 23 vitamins and minerals, including micronutrients (e.g., vitamin A, B vitamins, iron) and macrominerals (e.g., calcium, potassium, phosphorus, and magnesium) [169]. 

Therefore, the question remains if the present programs, donations, charity aid foundations, alliances on food and beverage fortification, which are frequently supported by well-known international premix producers, NGOs, and national regulations, supposedly tackling the malnutrition burden in SSA countries, may contribute towards a reduction in malnutrition or affect child malnutrition, which may be reflected in public health. 

**Figure 3:**
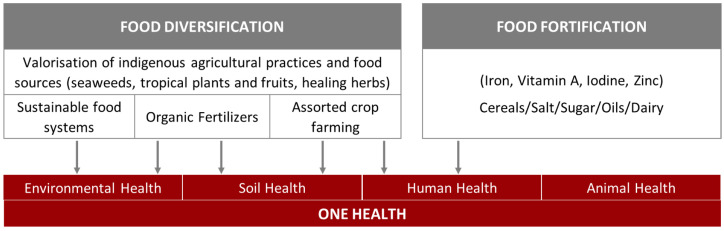
Fortification versus diversification, reducing micronutrient deficiencies within the holistic concept of One Health.

One Health is a collaborative, multi-sectorial, and trans-disciplinary approach towards achieving optimal public health outcomes, demanding surveillance, prevention, and mitigation, including water and food security/safety, and socioeconomic and cultural factors, which are influenced by food fortification (Figure 3) [170]. 

## 8. Fortification Strategies

Food fortification is not philanthropic or charitable, involves overall heavy costs, and can assume various shapes; the following three different forms may be accepted: (a) Mass fortification consists of the incorporation of micronutrients into edible products routinely ingested by consumers, such as cereals, oils and vegetable fats, milk, sugar, and condiments [171]; (b) Targeted fortification is the process of adding a large amount of micronutrients to foodstuffs for an abundance of the daily requirements designed for specific population subgroups, e.g., complementary foods for infants, for school feeding programs, and those for crisis circumstances [172]; and (c) Market-driven fortification is the process by which a food manufacturer intentionally decides to add one or more micronutrients to processed foods, with the purpose of attracting consumers and increasing sales [100]. 

Fortification practices and rules, now embedded in public food schemes, are different for each type of food fortification. Food fortification can be a useful tool in combatting micronutrient deficiencies. Its effectiveness depends on the skill and experience with which it is applied. Countries often face the challenge of determining which levels of nutrients are both efficacious and safe for the population at large. WHO’s updated guidelines for maize flour and corn meal fortification include some 10 micronutrients, but for iron, zinc and vitamin B_12_, less than 50% of the standards were met by the countries [173]. 

The lack of an overall program design, the prohibitive cost of the fortified foods’ system, technological demands, monitoring and evaluation difficulties, need to ensure that fortified foods are consumed in adequate amounts; however, ethical concerns impede the widespread adoption and effectiveness of this strategy in SSA. It is not a properly designed tool in all situations, and needs to be combined with other techniques in order to obtain the optimal result, therefore its use in SSA is debatable [143]. 

It is critical to identify all the factors involved in any given nutritional problem, namely food insecurity, inadequate dietary diversity, lack of nutrition education, and the state of local food processing are among the most significant factors adopted for the determination of the most appropriate strategy [174]. 

There are different considerations involved in the establishment of food fortification programs in developing countries as opposed to developed ones. The standards for fortified foods were published, for example, in India [175] but such guidelines for SSA are difficult to implement such as, for example, the provisions for the reference of the purity criteria of micronutrients. The identified vehicle must be consumed in roughly constant quantities throughout the year by a majority of the population, which is hard to achieve in SSA. Again, the facilitation of a rigidly controlled fortification process requires that the fortified food passes through a central point. Finally, acceptable ranges of fortification must be defined, but facilities are not readily available in SSA. 

Furthermore, the provision of micronutrients to those people who need the intervention must be determined while avoiding imbalanced or excessive intakes for other groups. The distribution of nutritional requirements and susceptibility to toxicity determine this risk/benefit calculation, and it seems that neither has been determined for most of the micronutrients [176].

Indeed, consumers in developed countries seek foods that promise health benefits, and the industry is providing just that and more [177]. But this scenario is not yet valid for most SSA countries, where subsistence rural farmers prevail, and food purchases are not yet influenced by a health concern, such as managing fat and cholesterol, salt and sugar, reducing risk of a disease, defying the aging process, or following doctor’s orders. In reality, some 80% of the SSA population attend traditional doctors and access to imported pharmaceuticals in SSA faces numerous challenges [178,179]. 

A notable disparity persists between the rural and urban areas and, indeed, food with a future will require that everyone should have access to a healthy and ecologically sustainable diet, which is still not a reality for all in SSA [1,180]. 

The marketing of fortified food products can be quite complex. The actual quantity of fortificants added to foods is another issue. Folic acid is a good case in point, since wheat flour contains folate but not folic acid, and it must be provided in effective amounts to women of childbearing age, without providing too much to the rest of the population. Hence, there is a need to have the same food product fortified with different amounts of a certain fortificant for different categories of consumers [35]. 

Similarly, while the general fortification of baby food is widely accepted as being an excellent way of adding micronutrients to their diet, it often cannot meet the growth and development needs of young children who ingest smaller amounts of foods and have higher micronutrient requirements. Hence, there is a need to determine how to provide the necessary micronutrients to those people who need them, while avoiding imbalanced or excessive intakes for other groups [181].

The majority of infant weaning foods available do not provide the nutrient density and diversity of taste and texture needed in this formative stage. Furthermore, children weaned on these products can develop unhealthy sweet tooth habits in the future, since 65% or more of these foods are sweetened [182]. 

Multinational firms have sometimes been responsible for health problems due to their fortified foods; for example, some consumers filed in 2017 a lawsuit against Nestlé, USA, for their bottled common groundwater and illegally mislabeled “spring”, “purified”, “mineral”, “sparkling”, water fortified with fluoride [183]. Similarly, there was a lawsuit against Gerber Products Company for their baby food and infant formula products fortified with fluoride [184]. 

In Nigeria, Nestlé introduced water enriched with zinc to overcome the national deficiency of this mineral, aimed at strengthening immunity [185,186], and Coca Cola released “vitaminwater^®^” with vitamins, electrolytes, and new flavor variants [187]. The need and suitability of such fortified beverage products are questionable, since they are not based on accurate and reliable information regarding the dietary habits, nutrition requirements, and nutrition status of target populations in the concerned SSA countries. 

The current levels of micronutrients added to maize meal and bread flour are considered unsatisfactory in South Africa. This is likely due to the inadequate incorporation of premixes at the mills, which affects the intake of the fortified product, and likely prevents the wanted reduction in micronutrient deficiencies anticipated from such a flour fortification program [188,189]. 

Such a case study clearly indicates that there is a need to conduct large-scale studies on crop composition and nutrient availability in the food matrix before creating dependence on imported micronutrient fortification, and to identify the right nutrients to be added to the right foods for populations most at risk for micronutrient deficiencies. 

The FDA in the USA and EFSA in Europe have always been against the practice of adding nutrients to “junk foods” just to make them seem healthy. But this does not stop food manufacturers, especially soft drink and juice manufacturers, from trying. 

Some authors even go as far as suggesting a role of soft drink industry in improving child health in SSA, advocating the fortification of soft drinks with micronutrients. In fact, instead of suggesting public health measures to counter the well-documented effects of soft drinks on rising rates of obesity or on tooth decay rates among children in developing countries, the authors propose that soft drink companies add vitamins and minerals to their drinks [190]. 

The Bill Gates Foundation partnered with Coca-Cola in Uganda and Kenya to increase production and distribution of mango and passion fruit juices as a way to stimulate production of these fruits and use them in Coca-Cola’s locally produced and sold fruit juices. Such a win-win partnership would also involve micronutrient fortification, empowering small farmers to increase productivity, improve crop quality and access to reliable markets critical to addressing global hunger and poverty [191]. 

The reality is that three out of four Americans do not consume the U.S. recommended daily allowance (RDA) for vitamins and nutrients [192]. So, the question is how one can expect Africans to be much better, despite having the SADC Minimum Standards for Food Fortification developed to provide member states with guidance on micronutrient additions to staple foods and condiments within the region [193]. Indeed, for RDA to accurately assist heterogeneous populations, they must be more inclusive of cultural differences and honor social practices to improve diet and reduce disparities [194].

In South Africa, there is a process of mandatory fortification (e.g., wheat, maize) which has been efficiently and effectively managed, supported by adequate monitoring and evaluation, food regulations, labelling and quality assurance. However, these procedures are not easy to implement in most of the other SSA countries [189]. Regarding cooking oil, some oil producers fortify on a voluntary basis, namely in Kenya since 2012 [195,196].

While the whole of SSA has 30 countries with mandatory wheat flour legislation, the Americas (North and South) have 35 countries with mandatory legislation for wheat. For salt, SSA as a whole has 45, and Asia has 35, while the Americas have 21 countries with mandatory legislation [197]. 

It is evident that the nutritional fortification of foods has been very effective in the past, at least immediately, in eliminating widespread nutritional deficiencies in developed countries. As consumer health awareness and scientific knowledge increase, there is a great need for the fortification policies to be re-evaluated. 

Furthermore, the amount of ingredients and nutraceuticals, such as ω-3 fatty acids, are not regulated, meaning that not only do consumers misperceive how much they should ingest, but manufacturers are not required to disclose how much or how little they are adding in their foods. Fortification has been portrayed as a silver bullet solution without considering critical perspectives and evidence, while private interests have been housed within national and international regulatory bodies.

Just because a food product is fortified does not mean it is healthy, and there are some limitations to this strategy. Fortified foods are usually heavily processed, and some micronutrients are sensitive to temperature, light, or oxygen, and get degraded when exposed or processed. The protection by micro encapsulation methods may not work for all micronutrients, namely ω-3 oils, which are quite easily degraded by oxidation [198]. 

Fortified foods interventions to reduce low birth weight and improve infant and child malnutrition were ranked only as “fair” [199]. Poor rural SSA populations have restricted access to fortified foods in the open markets due to low purchasing power and an immature distribution channel. 

In fact, SSA populations regularly eat more native maize-based foods, vegetables, cassava leaves, yams, herbs, spices, condiments, and tropical fruits, than 70% of North Americans, who, at the root of the obesity epidemic and many of its associated diseases, consume processed cereals, drink soda daily, have meat-rich diets that are too low in vegetables, fruit, and dairy, thereby falling short of meeting the dietary guidelines recommendations, and was where fortification started [200]. 

All trace elements are toxic if consumed at sufficiently high levels for long enough periods. The art of premixing consists in mixing all these ingredients homogeneously and limit the interactions, carefully selecting the raw materials. As the current literature on the stability of vitamins in vitamin premixes and vitamin/trace mineral premixes is limited [201], millers and premix suppliers often tend to add vitamins at dangerous levels, up to 100% of the recommended daily allowance in a single serving, possibly causing them to exceed the limit. In fact, fortification and enrichment of food products are not as innocent as they seem, since long-term health consequences of vitamins consumption are unknown [202]. 

Although fortification may increase the intake of vitamins and minerals, there is little evidence to suggest that adding nutrients (other than folic acid) may improve health [203]. Several experimental plans are available to reduce micronutrient deficiencies, but uncoordinated implementation of multiple interventions may result in excessive intakes [204].

In fact, there are growing concerns that fortifying and enriching foods may be harmful [205], and vitamin A was even suggested to be excluded from multivitamin supplements and food fortificants [206,207].

Supplements added to foods, even if consumed consistently, reveal less bioavailability, although these studies are complex since present methods do not allow us to distinguish nutrients in the experimental diet from the endogenous nutrients present in the body [208]. The immune system incorporates many organs and biological functions, and the so called “boost your immune system” with micronutrients makes very little scientific sense [209]. 

Fortified foods are more likely to spoil than are unfortified foods, and the addition of micronutrients creates an osmotic environment suitable for a wide range of microbiota, shaping the structure, composition, and function of the gut microbiome [210,211]. 

A major shift in our perception of food, agriculture, and nutrition is taking place in this era of globalization. Therefore, the high prevalence of malnutrition has become a good market for industrially produced food, which is technically designed for the corporate sector rather than farming communities [212]. 

The critics of mandatory food fortification call it out as ineffective, wasteful, and coercive. There is no evidence that it reduces the structural causes of micronutrients deficiencies and food insecurity. A mandatory policy, and scaling it up without adequate infrastructure and quality control, has concerning implications for the health and safety of vulnerable populations [213]. 

High levels of commitment are required from government agencies and food producers, along with extensive data necessary for effective program design and monitoring, and financial and infrastructure requirements for implementation of food fortification in SSA.

## 9. Other Strategies or Vehicles for Fortification

Bouillon cubes constitute a vehicle for fortification to overcome micronutrient deficiencies in SSA, providing flavor enhancement to savory foods. However, they possess a high sodium content, being a risk factor for hypertension and cardiovascular disease (CVD) [214]. Several multinational food companies are involved in the African bouillon market, including Maggi (Nestle), Knorr/Royco (Unilever), and Jumbo (GB Foods) [215].

Unilever favors salt reduction, having set benchmarks for sodium levels for different product groups. The formulation/modeling of benchmarks has been based on a total diet approach considering the contribution of the product group to overall daily salt intake [216,217].

Nestle’s Nutrition Profiling System (NNPS) has been developed for the reformulation of their products [218,219]. Micronutrient-fortified whole-grain maize meal (WGMM) in tropical Africa, employed in nutrition schemes and in packaged products [220], needs to have a long storage life due to challenges in distribution to rural areas. Hence, a thermal treatment is required to impede rancidity development in micronutrient-fortified WGMM. Hot-air drying of maize grain to 11.6% moisture substantially impedes rancidity development in fortified WGMM development as reported by Taylor et al. [221].

## 10. Policy Implications

The nutritional composition of Sub-Saharan foods and dietary patterns, based on the intake of healthier foods and nutrients, remain largely unknown, where the food culture dynamics have not received much attention [222]. 

The notion of “healthy food” has become omnipresent in medical, political, and media debate, as well as among the lay population. Indeed, most foods are, in fact, neither healthy nor unhealthy, but are just foods. Under the definition by the WHO of a healthy diet [223], depending on individual characteristics, cultural context, local availability of food, climatic and ecological conditions, dietary customs and preferences, most Africans definitely enjoy better, less processed, or unhealthy foods, exercise more and have poorer but healthier lifestyles than the average North American who eat unhealthy diets [224]. 

Nevertheless, the implementation of compulsory food fortification has been conducted almost exclusively in Africa, where mega entrepreneurs under several “initiatives” and “alliances” did not encounter a variety of scientific, technological, regulatory, and political barriers. In contrast, in Asian giants’ India and China, as in many developed countries, there has been general strong resistance to fortification. 

Through advocacy and framing activities, out-of-SSA influential mega-corporation producers of micronutrients have mostly led to conflicts of interest and well-documented policy interferences, with such producers even seeking to shape WHO policies through non-profit organizations or public–private partnerships, by planning and implementing, but rarely evaluating the outcomes of fortification. 

Fortification programs have encountered barriers associated with price, shops, and bazaars, and compulsory large-scale fortification programs have normally not been effective in delivering to the poorest areas due to imperfect food vehicle choices, market partition characterized by the predominance of informal traditional trade, as well as noncompliance by food and beverage manufacturing operations.

The lack of micronutrients can only be solved by dietary diversity, and food fortification with single micronutrients is just a band-aid solution. However, food fortification has been, for decades, largely a political public health intervention. The balance between business and public interests of non-government organizations, under the umbrella of the global food regulatory system of interest, has exerted substantial influence over food fortification. 

In SSA, national authorities have been promoting unwarranted mandatory food fortification laws, rarely evaluated or considered, which may result in adverse impacts on health and livelihoods.

## 11. Prediction of Excessive Intake of Micronutrients

Uncertainty encircling plausible fortuitous outcomes has led to apprehension, underlining the need to constantly monitor fortification programs for precise measures of their effect and the capacity to manage concerns as they arise. Some studies have shown opposite results regarding the lack of impact of food fortification programs and the guaranteeing of safe upper limits [225].

Presently in SSA, while trying to cover multiple nutrient interventions by different stakeholders, there is no effective surveillance or regular dietary surveys to help ensure that these interventions achieve their objectives. The food industry first ultra-processes staple foods, depriving them of essential micronutrients, and subsequently fortifies them, based on a claimed cost-effective public health intervention to reduce micronutrient deficiencies. This market-driven fortification does not, however, specify how to ensure its compatibility with indigenous foods and public health.

Success in food fortification is ensured by establishing the micronutrients’ Tolerable Upper Intake Level (UL) [226]. The WHO proposed a methodology for the calculation and definition of the safe upper limit in the Guidelines on Food Fortification with Micronutrients. However, high levels of consumption of the fortified food among the same population and utilization of multiple food vehicles remain a challenge [227]. In Cameroon, an excess of UL among children was reported following the consumption of multiple vitamin A-fortified vehicles such as sugar or wheat flour with edible oil [204,228].

A model has been based on the estimation of a Feasible Fortification Level (FFL), which was then used as a basis for the determination of the average, minimum, and maximum contents of the nutrients during production, taking into account the acceptable variation of the fortification process [35].

The usefulness of the application of mathematical modeling for the determination of nutritional requirement levels and provision of the basis for the rational formulation of complex mixtures of nutrients (diets) have been tested and developed to optimize accurate estimates for dietary nutrient levels, skipping the human trials [229]. 

## 12. Discussion and Concluding Remarks

The SSA people ingest plant foods unknown to the Western aid agencies and experts, while micronutrient interactions within different food matrix can impact on their absorption and bioavailability, which should be the basic priority for an adequate fortification policy. The ongoing corporate capture of the global nutrition business by hundreds of organizations threatens the achievement of food sovereignty and the full emancipation of women in the SSA region, bringing about industrialized food fortification that does not serve the SSA public health goals. 

Most aid organizations often have multiple purposes alongside their public health objectives, and they assist commercial companies in the creation of markets for their products. Statutory food safety regulators in SSA countries have made fortification mandatory but failed to avoid conflicts of interest and blurring information. This has opened up a new market for multinational premix companies and the mega food industry, while doing little to address the crisis of chronic under-nourishment and nothing to support family farmers. 

Since the intervention of fortification has not been monitored for decades, no evidence has been generated on its benefit or safety, while such a policy has been blindly accepted and law enforced. Perhaps the investment in alternative sustainable solutions based on indigenous foods would be more rewarding, improving food diversity and the access to basic public health care. In SSA countries, with the lack of evidence and scientific consensus, compulsory food fortification has been adopted, ignoring the role of native foods and of a balanced and diversified diet in addressing a variety of nutritional problems. To date, despite years of administration, it is not possible to estimate the outcomes of this intervention, such as mortality, morbidity, adverse effects, congenital anomalies, on follow-up as no trials have included these long-term outcomes.

Corporate-led fortification is becoming the main policy thrust, while holistic, balanced natural diets, produced and processed by the communities themselves, are not receiving the same attention. It is therefore uncertain whether food and beverage fortification reduces the risk of subclinical deficiencies, while the corroboration has been assessed as very low. 

Food fortification should support dietary improvement strategies as a complementary master plan and not as an alternative strategy, despite the fact that fortification has proven effective in treating single nutritional deficiencies in the past. From relative past success with fortification in other world regions, the key ingredients should be a formal cooperation among government bodies and the food industry, public education, initiatives based on epidemiological evaluations, and no direct conflicts of interest.

## Figures and Tables

**Table 1:** Some four main micronutrients are involved in different types of food fortification [97,110].

	Iodine	Iron	Vitamin A	Zinc
Condiments	Salt	Sauces (soy, fish)	Sugar	
Cereals		Wheat flour, corn flour, pasta, rice	Cereal flours	Cereals
Dairy products		Milk (powder and liquid)	Milk	
Fat and Oils			Oil, margarine	
Vegetables				Potatoes, beans

**Table 2:** A comparison of RAE (retinol activity equivalent) among different food sources [126].

Food	RAE per Serving (mcg)	Daily Value (%)
Animal liver 85 g	6600	731
Orange flesh sweet potato 100 g	1600	156
Spinach 60 g	570	64
Pumpkin 130 g	490	54
Papaya 100 g	756	32

**Table 3:** Iron levels in common African Foods.

African Food	Iron (mg/100 g FW)
Cowpea (*Vigna unguiculata*)	4.7
African spinach (*Amaranthus caudatus*)	5.9–12.7
Algae sirisiri (*Sesuvium portulacastrum*)	33.5
Alligator pepper (*Aframomum melegueta*)	37.8
Beef liver	6.7
Locust bean (*Leucaena leucocephala*)	33.6
Moringa (*Moringa oleifera*) leaves	28
Squash (*Pumpkin cucurbita*) leaves	3.2

## Data Availability

Not applicable.
